# Prevalence and factors associated with early initiation of breastfeeding among women in Moshi municipal, northern Tanzania

**DOI:** 10.1186/s12884-020-02966-0

**Published:** 2020-05-11

**Authors:** Hadija Y. Lyellu, Tamara H. Hussein, Margareta Wandel, Babill Stray-Pedersen, Melina Mgongo, Sia E. Msuya

**Affiliations:** 1 Institute of Public Health, Department of Community Health, Kilimanjaro Christian Medical University College (KCMUCo), Moshi, Tanzania; 2 Better Health for African Mother and Child (BHAMC), P.O. Box 8418, Moshi, Tanzania; 3 Institute of Basic Medical Sciences, Department of Nutrition, University of Oslo, Oslo, Norway; 4 Division of Gynaecology and Obstetrics, Oslo University Hospital, Rikshospitalet, Oslo, Norway; 5 Institute of Clinical Medicine, University of Oslo, Oslo, Norway; 6 Institute of Public Health, Department of Epidemiology & Biostatistics, Kilimanjaro Christian Medical University College (KCMUCo), Moshi, Tanzania; 7 Department of Community Medicine, Kilimanjaro Christian Medical Centre (KCMC), Moshi, Tanzania

**Keywords:** Breastfeeding, Early initiation of breastfeeding, Breastfeeding initiation, Prevalence, Factors, Tanzania

## Abstract

**Background:**

Early initiation of breastfeeding (EIBF) is a predetermining factor for exclusive breastfeeding, and thus a foundation for optimal breastfeeding practices. Rates of EIBF are low globally (42%) and in Tanzania (51%), yet few studies have been done on this issue in Tanzania. This study aimed to determine the prevalence and factors associated with early initiation of breastfeeding among women in northern Tanzania.

**Methodology:**

This study extracted information from a cohort of 536 women who were followed from 3rd trimester period October 2013 to December 2015 in Moshi municipal, northern Tanzania. The data for this paper was collected by the use of questionnaires at enrolment, delivery and 7 days after delivery. The analysis is based on data from 413 women for whom complete information was obtained. Log binomial regression analysis was used to determine factors associated with early initiation of breastfeeding.

**Results:**

The prevalence of EIBF was 83%. Overall, women had high knowledge on colostrum (94%), knowledge on exclusive breastfeeding (81%) and time of breastfeeding initiation (71%), but only 54% were counseled on breastfeeding during antenatal care. Knowledge on timely initiation of breastfeeding during pregnancy and vaginal delivery were associated with EIBF.

**Conclusion:**

Early initiation of breastfeeding is high (83%) in Moshi Municipal but still below the universal coverage recommended by WHO and UNICEF. There is missed opportunity by health facilities to counsel and support early initiation of breastfeeding given high antenatal and facility delivery in this setting. There is a need to evaluate health facility bottle necks to optimal support of early initiation of breastfeeding in Tanzania.

## Introduction

Early initiation of breastfeeding, defined as initiation of breastfeeding within 1 h after birth, is considered as one of the key interventions in ending preventable neonatal and child deaths and improving child survival [1–7]. Initiation of breastfeeding within the first hour of birth gives the best possible start in life. It increases the chances that newborns receives the first milk “colostrum”, that is rich in antibodies and nutrients, vital in protecting the newborn against infections [4, 6–10]. Immediate skin-to-skin contact which is important in facilitating early initiation of breastfeeding helps in regulation of newborns body temperature, and thus survival [1, 4]. Maternal advantages of early initiation of breastfeeding include stimulation of oxytocin release that helps uterus to contract hence reducing the risk of postpartum hemorrhage [11, 12]. It also enhances early bonding between mother and newborn and in establishing exclusive breastfeeding and continued breastfeeding [4, 5].

Despite neonatal, infant and maternal benefits of early initiation of breastfeeding, the rates are low. Globally only 42% of newborns were breastfed within 1 h of birth in 2017, an increase from 37% in 2005 [1]. In Asia early initiation of breastfeeding ranged from 32 to 40% [1, 5, 13]. In Sub Saharan Africa (SSA) the prevalence of early initiation of breastfeeding vary; 40% in West and Central Africa, 65% in Southern Africa, 62% in Kenya, 66% in Uganda and 80% in Rwanda [5, 13]. Tanzania however has a different trend to other East African countries. The prevalence of early initiation of breastfeeding has been fluctuating. In 2004, the rate was 59, 49% in 2010 and 51% in 2016 [14]. Thus identifying individual and facility factors that influences early initiation of breastfeeding is important.

Numerous factors have been associated with early initiation of breastfeeding. These include; cultural practices (like giving prelacteal feeding), antenatal care attendance, delivery at the Baby Friendly Hospital Initiative (BFHI) facility, mode of delivery, skilled birth attendant use and number of children [15–21]. Studies in South Asia, Nigeria and Tanzania have shown women who delivered at health facilities had 1.5–2 times higher odds of initiating breastfeeding within 1 h after birth than those delivered at home [14, 17, 18].

Globally different interventions have been implemented to protect, promote and support early initiation of breastfeeding. These includes the International Code for Marketing Breast-milk Substitute (1981), (BFHI) in 1991 and the global strategy for infant and young child feeding practices in 2000 [1, 5, 13].

Tanzania have adopted BFHI intervention for about 30 years, but the intervention is sub-optimally implemented. It is recoginsed that health care providers are key to the implementation of BFHI and to provide suportive skills to help mothers to position the baby during breastfeeding. However a study by Mlay (2011) showed that health care providers have inadequate skills to help and support mothers to initiate breastfeeding [22]. This is a missed opportunity since 65 and 91% of deliveries are occuring at the health facility at national and Kilimanjaro region respectively [14]. Accoding to Demographic and Health Survey (DHS) report, the rates of EIBF have been flactuating with a marked variations between regions ranging between 26 to 80% [14, 23, 24].

These variations in prevalence of early initiation of breastfeeding call for studies to understand the context regarding dynamics and factors associated with early initiation of breastfeeding at different settings. This study therefore aimed to determine prevalence and factors associated with early initiation of breastfeeding among women in Moshi Municipal, northern Tanzania. The information will contribute to improve EIBF practices which is one of the vital intervention to accelerate the achievement of Sustainable Development Goal (SDG) 3.2 of reducing preventable newborn deaths to 12 per 1000 live births by 2030 [5, 25].

## Methods

### Study site and design

The study was conducted in Moshi municipal council, situated in northern Tanzania. Moshi municipal is one of the seven districts of Kilimanjaro region. According to the Tanzania Census of 2012, Kilimanjaro region had a total population of about 1.64 million people and Moshi municipal council had estimated population of 185,000 and 57,000 women of reproductive age [26]. The economic activities in Moshi municipal council include business, tourism, small scale industries and small farming. By the end of 2017, Moshi municipal had 21 wards, 64 streets and 51 health facilities. The facilities are four hospitals, eight health centers, and rest dispensaries, owned by government, religious organizations and Non Governmental Organization (NGOs). There are 25 health facilities that provide reproductive and child health (RCH) services and delivery services are offered at eight facilities i.e. four hospitals and four government health centers.

The analysis of this study was part of larger cohort study that was conducted between October 2013 and December 2015 in two government primary health centers, i.e. Majengo and Pasua. The two clinics are the largest primary health centres in Moshi municipality; catch a huge population of pregnant women in Moshi urban and offer RCH and delivery services**.** Briefly, the study enrolled women in their third trimester of pregnancy and followed them at birth, at 7 days post delivery, and monthly up to 6 months after delivery, and thereafter every 3rd month up to 1 year post delivery [27, 28].

The larger study aimed to describe knowledge of optimal breastfeeding practices and intention to exclusive breastfeed during pregnancy and how they influence optimal breastfeeding practices of the infants after delivery [27]. The parent study also aimed to measure the rate of exclusive breastfeeding by using different methods of measurement [29]. This paper used the data that was collected from enrolment, delivery and up to 7 days after delivery.

### Study population and data collection procedures

#### Population

**Fig. 1:**
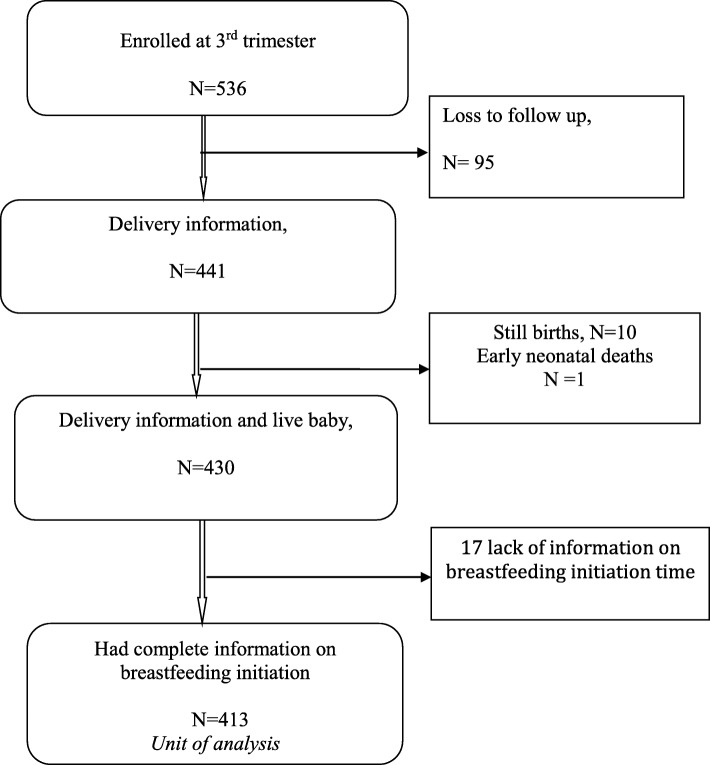
Follow up of participants

The study population for this paper was all women who were enrolled during pregnancy, were followed at birth and at 7 days post-delivery and had complete information on time of breastfeeding initiation. The parent study enrolled 536 pregnant women in their third trimester and 413 women had complete information on time of initiation of breastfeeding, thus involved in the analysis (Fig. 1).

#### Sample size

Sample size for this analysis was estimated by using the formula for precision

$$ N=Z2\ast P\left(1-P\ \right)/\varepsilon 2 $$

Where *N* is estimated minimum sample size; *Z* is confidence level at 95% (standard value is 1.96); *P* is proportion (prevalence of early initiation of breastfeeding in Kilimanjaro region of 73.7% by [22]; *ε* is precision at 95% CI = 0.05.

The minimum sample that was required for this study was 298 women. Addition of 10% for non-response gave a minimum sample of 328 pregnant women.

#### Study procedures

Before enrollment, pregnant women were informed about the study aims and follow up procedures. The women who gave an informed consent and who reported they would be in Moshi for at least 9 months after delivery were enrolled in the study. After consenting, face- to-face interviews were conducted using a questionnaire by trained nurses/ junior doctors. The interviews were in Swahili.

At enrollment the questionnaire was used and it collected information on: socio-demographic and economic characteristics, partners’ characteristics, reproductive health information including parity, timing and frequency of antenatal attendance, information on counseling on breastfeeding or infant feeding during antenatal visits, and type of advice on breastfeeding given. Information on breastfeeding knowledge was also collected and it included knowledge on colostrum, pre-lacteal feeding, knowledge on optimal time to initiate breastfeeding and knowledge on exclusive breastfeeding definition and duration.

At delivery a standardized tool was used to collect information on; place of delivery, mode of delivery, sex, weight and length of the baby, if the baby was term or preterm and feeding/ breastfeeding information. The tool at 7 days visit included information on exclusive breastfeeding; any breastfeeding problems (mastitis, engorgement, cracked nipple) and ascertained information on time of breastfeeding initiation.

### Data analysis

Data was extracted and analysed by using SPSS version 23. Cleaning was conducted using frequencies and all the entries with missing values for the key outcome variable i.e. early initiation of breast feeding were removed. Descriptive statistics were used to summarize characteristics of study participants: continuous variables were summarized by using mean and standard deviation (SD) and categorical variables were summaries by using frequency and proportions. Odds ratio and 95% confidence interval were used to measure the strength of association between early initiation of breastfeeding and independent variables. Multivariable log-binomial regression was used to determine the factors independently associated with early initiation of breastfeeding. The *p* value of less than 0.05 was considered as statistical significant.

### Categorization of variables

Early initiation of breast feeding is defined by WHO as initiation of breastfeeding within 1 h of birth [1]. Categorization of breastfeeding initiation was as follows: within 1 h after birth; 2–23 h; and 24 or more hours after birth. Age of participants which was collected as numerical variable and was categorized (< 20, 20–34, and 35–49). Marital status was categorized as those in union vs. not in union, and parity as (first and second or more).

Categorization of knowledge of early initiation of breastfeeding practices was measured through a series of questions and those participate who mentioned a child should be put on breast within an hour after birth were categorized as having knowledge on early initiation of breastfeeding. Knowledge on colostrum and its advantages was assessed and women who reported that it is important that the newborn should be given colostrum were categorized as having knowledge.

## Results

### Background characteristics of the participants

#### Socio demographic characteristics of the women

The mean age of the 413 participants was 26.3 (SD 5.6) years. Most of the women were married/cohabiting 371 (89.8%), and were not employed in formal sector 371 (89.8%). Nearly one third of the women 144 (34.9%) had an income per month of less or equal to 60,000 Tanzanian shillings (≤ 30 USD). Other socio demographic characteristics are shown in (Table 1).

**Table 1:** Socio-demographic characteristics of women in Moshi municipal by clinics (*N* = 413)

Characteristics	Overall Frequency (%)	Health Facility	
		Pasua (***n*** = 228) Frequency (%)	Majengo (***n*** = 185) Frequency (%)
**Maternal age (years)**			
< 20	39 (9.4)	27 (11.8)	12 (6.5)
20–34	338 (81.9)	187 (82.0)	151 (81.6)
≥ 35	36 (8.7)	14 (6.2)	22 (11.9)
**Religion**			
Christian	234 (56.7)	92 (40.4)	142 (76.8)
Muslim	179 (43.3)	136 (59.6)	43 (23.2)
**Marital status**			
Single/widow/Separated	42 (10.1)	23 (10.1)	19 (10.3)
Married/Cohabiting	371 (89.8)	205 (89.9)	166 (89.7)
**Education status**			
None and primary	254 (61.5)	153 (67.1)	101 (54.6)
Secondary and above	159 (38.5)	75 (32.9)	84 (45.4)
**Occupation status (*N*** **= 410)**			
Self employed	371 (90.5)	211 (93.3)	160 (87.0)
Employed	39 (9.5)	15 (6.6)	24 (13.0)
Maternal income* (*N* **= 284)**			
≤ 60,000Tshs	144 (50.7)	92 (53.2)	52 (46.8)
> 60,000Tshs	140 (49.3)	81 (46.8)	59 (53.2)
**Alcohol use**			
Yes	61 (14.8)	16 (7.0)	45 (24.3)
No	352 (85.2)	212 (93.0)	140 (75.7)
**Partners information’s**			
**Education level (*N*** **= 408)**			
None and primary	221 (54.2)	130 (57.8)	91 (49.7)
Secondary and above	187 (45.8)	95 (42.2)	92 (50.3)
**Occupation status (*N*** **= 412)**			
Self employed	246 (59.7)	143 (63.0)	103 (55.7)
Employed	166 (40.3)	84 (37.0)	82 (44.3)
**Child information**			
**Sex of the child (*N*** **= 409)**			
Male	201 (49.1)	107 (47.8)	94 (50.8)
Female	208 (50.9)	117 (52.2)	91 (49.2)

*At the time of the study 1 USD = 2000 TZS

#### Reproductive characteristics, knowledge of breastfeeding and practices

The majority of the 413 women (99%) delivered at the health facility and mode of delivery was vaginal delivery (88%), Table 2. The majority of the women (94.7%) had good knowledge on colostrum giving and its importance and 71% of the women had knowledge that breastfeeding should be started within 1 h after birth. About 94% of the 413 women gave colostrum and 7% reported to have given pre-lacteal feeds. Missed opportunity for counseling was reported, as only 54% of the women reported to have received counseling on breastfeeding issues during pregnancy.

**Table 2:** Reproductive health characteristics, knowledge on breastfeeding and practices among 413 women in Moshi municipal

Knowledge and reproductive health characteristics	Overall Frequency (%)	Health Facility	
		Pasua (***n*** = 228) Frequency (%)	Majengo (***n*** = 185) Frequency (%)
**Number of ANC visit (*N*** **= 407)**			
< 4 visits	274 (67.3)	173 (75.9)	101 (56.4)
≥ 4 visits	133 (32.7)	55 (24.1)	78 (43.6)
**Parity (*N*** **= 268)**			
Prime	138 (51.5)	76 (47.5)	62 (57.4)
Multipara	130 (48.5)	84 (52.5)	46 (42.6)
**Counseled on BF/infant feeding at ANC visit**			
No	189 (45.8)	95 (41.7)	94 (50.8)
Yes	224 (54.2)	133 (58.3)	91 (49.2)
**Type of advice given**^**a**^			
Correct positioning (Yes)	117 (28.3)	56 (24.6)	61 (33.0)
Correct attachment (Yes)	92 (22.3)	44 (19.3)	48 (25.9)
Solving BF problem (Yes)	41 (9.9)	38 (16.7)	3 (1.6)
Colostrum giving (Yes)	88 (21.3)	53 (23.2)	35 (18.9)
Exclusive breastfeeding only (Yes)	185 (44.8)	107 (46.9)	78 (42.2)
**Knowledge on time of breastfeeding initiation**			
After 1 h of delivery (delay)	27 (6.5)	13 (5.7)	14 (7.6)
Within 1 h after delivery (early)	293 (71.0)	166 (72.8)	127 (68.6)
Don’t know	93 (22.5)	49 (21.5)	44 (23.8)
**Knowledge on colostrum giving**			
No	12 (2.9)	9 (3.9)	3 (1.6)
Yes	391 (94.7)	216 (94.7)	175 (94.6)
Don’t know	10 (2.4)	3 (1.4)	7 (3.8)
*Information collected after delivery*			
**Place of delivery (*N*** **= 410)**			
Home	3 (0.8)	2 (0.9)	1 (0.5)
Health institution	407 (99.2)	223 (99.1)	184 (99.5)
**Mode of delivery (*N*** **= 408)**			
Caesarean Section	46 (11.3)	21 (9.3)	25 (13.7)
Vaginal delivery	362 (88.7)	205 (90.7)	157 (86.3)
**Colostrum giving practice (*N*** **= 412)**			
No	25 (6.1)	18 (7.9)	7 (3.8)
Yes	387 (93.9)	209 (92.1)	178 (96.2)
**Time of BF initiation**			
Within 1 h after birth	343 (83.1)	205 (89.9)	138 (74.6)
2–23 h after birth	51 (12.3)	22 (9.7)	29 (15.7)
≥ 24 h after birth	19 (4.6)	1 (0.4)	18 (9.7)
**Pre-lacteal feeds practice**			
Provided	28 (6.8)	5 (2.2)	23 (12.4)
Not provided	385 (93.2)	223 (97.8)	162 (87.6)

a: Multiple answered were recorded with Yes or No answer.

### Prevalence of early initiation of breastfeeding

Of the 413, the prevalence of early initiation of breastfeeding was 83.1%, (Table 2). Nearly 5% of the newborns were breastfed after 24 h of birth.

### Factors associated with early initiation of breastfeeding

Table 3 depicts the association of socio-demographic characteristics with early initiation of breastfeeding.

**Table 3:** Association between socio-demographic factors and early initiation of breastfeeding among women in Moshi municipal, (*N* = 413)

Variables	Total (N)	EIBF n (%)	Crude OR [95% CI]	Adjusted OR [95% CI]
**Maternal age (years)**				
< 20	39	36 (92.3)	1.11 [1.01–1.23]	0.99 [0.63–1.54]
20–34	338	280 (82.8)	1.0	1.0
≥ 35	36	27 (75.0)	0.91 [0.75–1.10]	0.75 [0.24–2.39]
**Marital status**				
Unmarried	42	33 (78.6)	0.90 [0.4–2.0]	0.93 [0.41–2.14]
Married/cohabiting	371	310 (83.6)	1.0	1.0
**Education status**				
None and primary	254	209 (82.3)	1.0	1.0
Secondary and above	159	134 (84.3)	1.02 [0.94–1.12]	0.95 [0.42–1.24]
**Maternal income**				
≤ 60,000Tshs	144	119 (82.6)	1.0	1.0
> 60,000Tshs	140	119 (85.0)	1.19 [0.63–2.24]	1.31 [0.61–2.82]
**Partner education level**				
None and primary	221	180 (81.5)	1.0	1.0
Secondary and above	187	159 (85.0)	1.29 [0.76–2.19]	1.82 [0.84–3.96]
**Partner occupation status**				
Self employed	246	204 (82.9)	1.0	1.0
Employed	166	138 (83.1)	1.01 [0.60–1.71]	0.73 [0.36–1.49]
**Health facility enrolled**				
Pasua	228	205 (89.9)	1.0	1.0
Majengo	185	138 (74.6)	0.73 [0.68–0.80]	0.93 [0.76–1.15]

Adjusted for maternal age, marital status, mother’s education level, mother’s income level, partner education level, partner occupation status and health facility women enrolled

In unadjusted model clinic of enrolment was significantly associated with EIBF, but the results were not significant in adjusted model. Other factors like age of the mother, marital status, mother’s education level, mother’s occupation, mother’s income level, and partner education level and partner occupation status were assessed but they were not significantly associated with early initiation of breastfeeding.

The association between early initiation of breastfeeding with knowledge, breastfeeding practices and reproductive health factors is shown in Table 4. In multivariable regression analysis, Women who had caesarean section had 93% less odds of initiating breastfeeding early compared to women who gave birth by vaginal delivery (Adjusted odds ratio (AOR) = 0.07 (0.03–0.16). Women who had knowledge on time of breastfeeding initiation during pregnancy had 3 times more higher odds to initiate breastfeeding early compared to others (Adjusted OR = 3.01 (1.56–5.81).

**Table 4:** Multivariable log-binomial regression to determine association between reproductive health factors, knowledge of breastfeeding and practices with early initiation of breastfeeding in Moshi municipal (*N* = 413)

Variables	Total (N)	EIBF n (%)	Crude OR [95% CI]	Adjusted OR [95% CI]
**Number of ANC visit**				
< 4 visits	274	235 (85.8)	1.0	1.0
≥ 4 visits	133	105 (79.0)	0.79 [0.72–1.06]	0.96 [0.83–1.10]
**Parity**				
Prime	138	113 (81.9)	1.0	1.0
Multipara	130	108 (83.1)	1.01 [0.91–1.13]	1.02 [0.91–1.14]
**Type of advice given during ANC**^**a**^				
Correct positioning (Yes)	117	97 (82.9)	1.01 [0.89–1.14]	0.92 [0.80–1.07]
Correct attachment (Yes)	92	76 (82.6)	1.00 [0.88–1.13]	0.97 [0.86–1.11]
Colostrum giving (Yes)	88	74 (84.1)	1.03 [0.91–1.17]	1.01 [0.86–1.13]
EBF only (Yes)	185	153 (82.7)	1.02 [0.85–1.22]	0.39 [0.19–0.80]
**Knowledge on timely BF initiation during pregnancy**				
Others ^b^	66	46 (69.7)	1.0	1.0
Within 1 h (timely)	347	297 (85.6)	2.59 [1.41–4.74]	3.01 [1.56–5.81]
***Information collected after delivery***				
**Mode of delivery**				
Caesarean section	46	17 (37.0)	0.08 [0.04–0.07]	0.07 [0.03–0.16]
Vaginal delivery	362	321 (88.7)	1.0	1.0

^a^: Yes vs No

^b^:others (mentioned initiation after 1 h, I don’t know)

Adjusted for number of antenatal care (ANC) visits, parity, type of advice given during ANC, knowledge of early initiation of BF, and mode of delivery.

## Discussion

In this study, 83% of the women breastfed their newborns within the 1 h after birth. Mode of delivery and mode of delivery were the independent factors associated with of early initiation of breastfeeding.

The prevalence of early initiation of breastfeeding observed in the current study of 83% is higher than the proportions reported in earlier studies in Kilimanjaro region. Over time proportion of women initiating breastfeeding within an hour of birth increased from 70% in 2002–06 [30] to 77% in 2010–11 [31]. Tanzania Demographic and Health Survey of 2015–16 reported the national prevalence of early initiation of breastfeeding of 51%, with wide regional variations; ranging from 26% in Simiyu region to 80% in Tanga region. Kilimanjaro region had prevalence of 74% according to the TDHS of 2015/16. It seems over time women in the region are adapting positive practices of early initiation of breastfeeding. In this study, high early initiating of breastfeeding was paralleled with overall high knowledge on colostrum and its importance (94.7%), knowledge on definition and time for exclusive breastfeeding (81%) and knowledge that newborns should start to be breastfeeding within 1 h (71.0%) [27]. Community and facility awareness and education campaigns are still needed, as WHO and UNICEF recommend that every newborn should be put on breast within the first hour after birth as “it gives them the best chance to survive” [1, 4].

Women who had correct knowledge on time of breastfeeding initiation during pregnancy, had higher odds of initiating breastfeeding within 1 h after birth. Studies in Ethiopia, revealed that those women who receive proper counseling on breastfeeding during pregnant are more likely to have early breastfeeding initiation [19, 32]. Similar findings were also observed in Brazil [15], showing the need to improve counseling on optimal breastfeeding practices among women during antennal period.

There was however, missed opportunity to improve early initiation of breastfeeding and overall optimal breastfeeding practices in this setting. Despite the fact there is universal antenatal care attendance at least once and high health facility delivery (90%) in TDHS and 99% in this study, only 54% of the women reported to have received counseling on breastfeeding or on infant feeding during pregnancy [27]. In Tanzania, more women are attending for pregnancy care and are delivering at the health facilities with skilled providers. Proportion of births attended by skilled providers has increased from 46% in 2004–05, 51% in 2010 and 64% in 2015–16 as reported by Demographic and Health survey. This is in contrast to early initiation of breastfeeding where rates have declined from 59% in 2004–05, to 49% in 2010 and slightly rose to 51% in 2015–16 survey [14]. Health providers need to use the opportunity to have intensive and sustained education and communication campaigns on optimal breastfeeding practices including early initiation of breastfeeding when meeting women during pregnancy, delivery and postnatal care.

Providers should be educated to understand the critical role they have in reducing mortality among neonates. By using the critical moment to start breastfeeding, they are contributing in reducing neonatal mortality, infection related mortality and child mortality. Further, neonatal mortality accounts for 40 - 47% of child mortality [1, 5], therefore improving optimal breastfeeding practices especially early initiation of breastfeeding may be one of key interventions in countries with high neonatal mortality like Tanzania (21 per 1000 live births) and help to accelerate the achievement of SDG 3.2 of reducing preventable newborn and child deaths deaths to 12 and 25 per 1000 live births by 2030 [13, 33].

Mode of delivery was a strong predictor of early initiation of breastfeeding in this study. Women with vaginal delivery were three times more likely to practice early initiation of breastfeeding compared to caesarean section. Similar observations have been noted in studies conducted in Ethiopia, Nigeria, India, Brazil as well as in Tanzania which reported that women who had ceasarian section had delay initiation of breastfeeding compared to those with vaginal delivery [17, 18, 20, 21, 34]. Women who have undergone ceasarian section may have experienced maternal pain, fear and stress, fatique and prolonged recovery [35]. Furthermore, infant born by Ceasarian section are more likely to have respiratory distress that might cause a newborn to be taken to intensive care unit consequently being separated from mother [16] while the reasons above might offer an explanation, each individual facility should have policies and interventions in place that would try to initiate breastfeeding in optimal manner even among women who have undergone caesarean section.

There was lack of association between early initiation of breastfeeding and sex of the baby, residency, and wealth of the household or parental education level. This is similar to demographic and health survey results of 2015–16 in Tanzania and by WHO & UNICEF of 2018. It is in contrast to other studies in India where older women (≥ 35 years) were less likely to initiate breastfeeding early than younger women [36] and in Ethiopia where mothers from wealthier households were more likely to commence breastfeeding early as compared to mothers from poorest household [17]. However in all studies similar to this, very few individual factors influenced early initiation of breastfeeding. It points to the fact that health facility factors would be more important and studies are needed to understand the gaps and dynamics of how facilities handle essential newborn care [37]. Globally, 70% of the women deliver at health facilities, and facilities are with women in the first hour after delivery hence more roles to play in improving initiation of breastfeeding at optimal moments/ time.

## Strength and limitations

Early initiation of breastfeeding information’s was collected based on women self-report; this may lead to over reporting of the behavior. However the interviews were conducted within 24 h of delivery and thus minimized recall bias. Health facility factors like training of providers in breastfeeding or years of experience that may have influenced counselling and support for EIBF at facilty level were not collected in this study.

However, the study has strength of being prospectively in nature that allowed mothers to be enrolled in the study before delivery and to be visited shortly after delivery which helps to overcome recall bias. While the study was health facility based, proportion of women who delivered at health facilities was nearly universal (99%), therefore the results can be generalized to women of reproductive age in the municipal.

## Conclusion

Prevalence of early initiation of breastfeeding in Moshi Municipal was high (83.1%) but was below WHO recomandation of 90%. Knowledge on correct time of breastfeeding initiation during pregnancy and mode of delivery were the independent predictors of early initiation of breastfeeding.

In order to improve the rate of early initition of breastfeeding in Moshi Municipal, District Health Management team should strengthening interventions on promotion and supporting early initiation of breastfeeding. Counselling on optimal BF and EIBF should be strengthened and promoted. Given high facility deliveries, immediate support after delivery on attachment and positioning might help to improve early initiation of breastfeeding. Moreover, more reseach especially qualitative should be done in Majengo health center to explore more on why most women delayed to initiate breastfeeding as recommended by WHO.

## Data Availability

The dataset analysed for the current study is available from the corresponding author on reasonable request.

## Abbreviations

ANC: Antenatal care
BF: Breastfeeding
BFHI: Baby Friendly Hospital Iniative
EBF: Exclusive Breastfeeding
EIBF: Early Initiation of Breastfeeding
RCH: Reproductive and Child Health
SDGs: Sustainable Development Goals
WHO: World Health Organization
